# The Annual Report of the Buffalo State Asylum for the Insane

**Published:** 1883-05

**Authors:** 


### THE ANNUAL REPORT OF THE BUFFALO STATE ASYLUM FOR THE INSANE.

Through the courtesy of Dr. Andrews, we have been favored with the twelfth annual report of the managers of the Buffalo State Asylum for the Insane, including the report of the Superintendent which gives valuable statistical information in regard to the work of this excellent institution for the year 1882. From a casual examination of the report, we find that there has been treated during the year at the asylum 429 patients; the daily average of patients has been 212$\frac{2}{9}$; the number admitted during the year 273, and the ratio of recoveries to the number admitted has been twenty per cent. The total amount of receipts from all sources has been $70,519.05, and the total expenditures $68,280.44, leaving a balance on hand of $2,238.61.

The statistical data as to the causes of insanity and of hereditary transmission are very instructive. We find intemperance an important factor in the causation of mental aberration, while grief, anxiety, overwork and loss of sleep, some of which are conditions peculiar to the high-pressure life of the American people, furnish a large percentage of cases.

The report furnishes the following instances of heredity:

In three instances a brother and sister were admitted, in one instance two brothers. The following were some of the most marked cases of heredity: a paternal grandfather, two paternal uncles and a brother; maternal uncles and two brothers; father, brother and sister, maternal grandfather and two maternal aunts; mother and two maternal aunts; and both father and mother.

To our readers, however, the most interesting portion of the report will be found in the following extract, which furnishes the experience of the medical staff of the asylum in the use of hyoscyamine:

As regards the remedies found useful as palliatives in certain states of the disease we desire to record our experience in the use of hyoscyamine. We have found it of great assistance in controlling outbursts of boisterousness and violence which characterize the disease. We have seen no ill effect and nothing like the unpleasant and dangerous symptoms which have been alluded to by some experimenters in the use of the drug. These we believe to have arisen from the unnecessary large doses taken. If the medicine is used at first in minute doses, and then gradually carried up to the point of control only, the desired result will be attained. It is neither necessary nor desirable to prostrate the patient and produce the full physiological effect. The dose of one-twentieth of a grain increased to one-fourth as needed will usually be sufficient. Comparative quiet and repose follow the smaller doses. We have found beneficial results in combining it with morphia and the bromides separately in doses of from one-eighth to three-eighths of the former and x to xxx grains of the latter. In this way the one-twelfth of a grain of hyoscyamine is often sufficient to accomplish the purpose as stated above. The combination is also useful as a hypnotic.

This dose administered in the morning gives several hours of quiet, and ordinarily need not be repeated till bed-time. In some instances hyoscyamine is contra-indicated; when it does not control the excitement and disturbs the appetite it should not be continued, as loss of flesh and injury to the general physical condition result. Trial of the drug only furnishes the criterion of its value in each individual case. In delirium tremens we have employed it in a limited number of cases. A few drops of the solution from the one-twelfth to the one-sixth of a grain given in milk punch proved sufficient to control muscular agitation and procure sleep. We cannot recommend this to the exclusion of chloral and the bromides, opium and other remedies which have an established value in the treatment of the disease, but desire to call attention to it as an additional therapeutic measure. In combination we have given it only by the mouth, while recognizing the fact that it may be decomposed by the secretions of the stomach; this has seldom occurred. The physiological effects have been studied by several observers and their similarity to those of atropia noted, and by some considered identical. The effects of a full dose are rapidly produced and may be briefly stated as follows: Increase of pulse, decrease of respirations, flushing of face, increase of external temperature, loss of motor power, due to paralysis of the motor nerves, profound sleep lasting for some hours. The secondary effects are a numerical decrease in the pulse beats and respiration and a fall of temperature. The most unpleasant effects of the medicine are the dryness of the fauces, the dilatation of the pupils which produces disturbance of vision, and the loss of muscular power. These are sometimes appreciated by the patients and are referred to by them in the order in which they are recorded here. They are rarely so marked as to render the discontinuance of the drug necessary.

It will be observed that the asylum is conducted, in its professional and scientific work, with the most thorough and painstaking efforts and care; that the patients are given the benefit of the best treatment, disciplinary, medicinal, as well as moral, which medical science can provide, and that the result of the work is creditable alike to the Superintendent, Dr. Andrews, and his able assistants, Drs. Granger and Crego, as well as to the State which fosters and sustains it. We are gratified to be able to give our warmest endorsement to the asylum, and to the excellent work it is doing for science and for humanity.

